# Comparative Aquatic Risk of Three Glyphosate–Based Herbicides Using Early-Stage Development of *Clarias gariepinus* (Burchell, 1822)

**DOI:** 10.1155/jt/9102995

**Published:** 2025-04-01

**Authors:** Chukwuma Okereke Ofor, Elizabeth Ogechukwu Uzochukwu, Chima Emmanuel Akudike, Paul Chinedu Onuoha

**Affiliations:** Department of Fisheries and Aquatic Resources Management, College of Natural Resources and Environmental Management, Michael Okpara University of Agriculture, P. M. B. 7267, Umudike, Abia, Nigeria

**Keywords:** cleavage, embryogenesis, fertilization rate, hatching rate, lowest significant effect concentration, microconcentration, oxidative stress

## Abstract

*Clarias gariepinus'* (Burchell, 1822) early-stage development was assessed in microconcentrations of glyphosate-based herbicides (GBHs), Forceup, Roundup, and Uproot. Using the default ecological trigger value of 0.37 mg L^−1^ of glyphosate as a reference, herbicides were diluted to microconcentrations containing 0.006, 0.013, 0.025, 0.05, and 0.10 ([v/v] %) of herbicide using borehole water, which served as control. Concentrations and control were replicated three times. Fertilization (%), time to morula formation and to commencement of hatching (minutes), hatching (% fertilized eggs), and 96-h larval survival (% hatched larvae) in microconcentrations were monitored. Within formulation, concentration significantly affected fertilization and hatching rates (*p* < 0.001), time to morula formation and hatching, and 96-h larval survival ([*χ*^2^] 5 = 16,648, *p* = 0.010; [Kruskal–Wallis H test]). Morula formation, fertilization, hatching, and larval survival rates were significantly affected by formulation in Concentrations 2 and 3, while fertilization rate was significantly affected at all concentrations ([*χ*^2^] 3 = 6.49, *p* = 0.039). The glyphosate ecological trigger value of 0.37 mg L^−1^ as well as the recommended application rate of Roundup Proactive in aquatic and riparian environments of 0.32% (v/v) are higher than the lowest significant effect concentrations of the herbicides. Reactive oxygen species (ROS) and superoxide dismutase (SOD) in control embryos, were higher but not significantly, than levels in freshly stripped eggs (*p* > 0.05) (Mann–Whitney *U* test). Early-stage development was normal in controls, suggesting a balance between ROS and SOD. This was, however upset in treatments, leading to deleterious effects on early-stage development. GBHs pose a greater risk to fish reproduction, varying in severity with the formulation. This should be considered in regulations for their use in aquatic and riparian environments, balancing herbicide effectiveness with the risk of aquatic toxicity.

## 1. Introduction

Glyphosate (N-[phosphonomethyl] glycine) is a postemergent, systemic, broad-spectrum herbicide and is one of the most commonly used herbicides [1, 2]. It is a pervasive environmental contaminant [3, 4]. Glyphosate achieves the herbicidal effect by inhibiting the enzymatic activity of 5-enolpyruvylshikimate-3-phosphate (EPSP) synthase, which disrupts the synthesis of some important metabolites in plants [5, 6]. Since EPSP is absent in animals, glyphosate was considered relatively safe on animals [7]. Recently, glyphosate toxicity in fish embryos [8] and larvae [9] has been reported.

The use of glyphosate necessitates the inclusion of additives that enhance its herbicidal effectiveness. The additives used vary between glyphosate-based herbicide (GBH) brands [10]. The brands used locally include Bushfire, Clearweed, Forceup, Mulsate, Sarosate, Sunphosate, Roundup, Uproot, and Vinash and are available as 360 g of glyphosate L^−l^ (in the form of 480 g L^−1^ glyphosate–isopropylamine salt) soluble liquid. Fully formulated GBHs are also commonly reported as being more toxic than the active ingredient or surfactant alone, for instance on *Drosophila melanogaster* [11]. However, Asnicar et al. [12] reported a contrary effect on sea urchin larvae.

Though GBHs are also used for aquatic weed management [13], most studies on aquatic toxicities of GBHs used fish juveniles or adults as test subjects. However, fish in early stages have a higher sensitivity to environmental pollutants than adults [14]. This is due to their higher surface-to-volume ratio resulting in a higher capacity to absorb, as well as a lower capacity to metabolize, chemical substances [12]. There is thus a need to ascertain the aquatic toxicities of these formulations using fish early stages, in order to provide a more sensitive assessment of their toxicities and a guide for their environmentally safe usage. This is necessary because of the increasing cultivation of glyphosate-tolerant crops leading to the growing use of GBHs [15, 16]. Some of the effects of GBHs on the developmental stages of other phyla include delay in the hatching of earthworm cocoons in the soil [17], extension of the larval period of American toads [18], and an increase in reactive oxygen species (ROS) and cell death of zebrafish larvae [19].

ROS include superoxide (O_2_^−•^), hydrogen peroxide (H_2_O_2_), hydroxyl (OH^−^), hydroperoxyl (HOO^•^), and peroxyl radicals (ROO^•^). ROS damage cellular macromolecules including proteins and lipids, with cellular proteins being primary targets for ROS attack [20]. Superoxide dismutase (SOD) is a group of metalloenzymes existing in all aerobic organisms susceptible to oxidative stress from ROS [21]. They are considered the first line of defense against the toxic effects of ROS. SOD activity is therefore, a marker of oxidative stress. SOD catalyzes the dismutation of superoxide molecules to molecular oxygen [22], preventing further damage.

The aim of this study is to assess the ecotoxicity of three commonly used GBHs on the early development of an ecologically and economically important freshwater clariid, *Clarias gariepinus* (Burchell, 1822), using environmentally relevant concentrations. The objectives are to determine the effect of microconcentrations of the herbicides, on *C. gariepinus* egg fertilization rate, progression of embryogenesis of the fertilized eggs, egg hatching rate, survival rate of the resulting larvae, and oxidative stress markers in eggs and larvae.

## 2. Materials and Methods

### 2.1. Herbicide Solutions

Glyphosate and GBHs are endocrine and energy disruptors [23]. Roundup, Forceup, and Uproot (glyphosate content 360 mg L^−1^) (manufacturers' information), which are representative of locally-used GBHs, were purchased from local agrochemical stores. The herbicides contained a water-based concentrate of 360g acid equivalent glyphosate (480 mg active ingredient) L^−1^ (as isopropylamine salt of N-[phosphonomethyl glycine]). The default ecological trigger value of glyphosate protecting 99% of freshwater species is 0.37 mg L^−1^ [24]. Using this as a reference, the herbicides were serially diluted to yield 0.023 mg L^−1^, 0.05 mg L^−1^, 0.09 mg L^−1^, 0.18 mg L^−1^, and 0.36 mg L^−1^ of glyphosate using borehole water as a diluent. The resulting concentrations of the full formulations were 0.006%, 0.013%, 0.025%, 0.05%, and 0.1% (v/v), respectively. These were designated Treatments 1, 2, 3, 4, and 5, respectively. The control contained 0 mg L^−1^ herbicide. The solutions were used in a bioassay to determine the effect of the concentrations on fertilization of *C. gariepinus* eggs, progression of the embryo through cleavage and hatching, and 96-h larval survival. Borehole water was used as a control. Concentrations and control were replicated three times.

### 2.2. Egg and Sperm Procurement

Farm-raised, pubertal, broodstock was used for egg and sperm procurement. Female *C. gariepinus* were artificially induced using Ovaprim. Eggs were dry-stripped and fertilized as per Ofor and Oke [25]. Stripped eggs from the three females were pooled. Likewise, milt from the three males was pooled. The mean number of eggs in one g egg mass was determined to be 760 ± 17 eggs. Pooled eggs and sperm were mixed. One gram of egg–sperm mix was then weighed in petri dishes.

### 2.3. Bioassay

Ten mL of each herbicide treatment was pipetted into petri dishes and used for sperm and egg activation. The herbicide–sperm–egg mix was stirred for 1 minute. The eggs were then placed in fresh preparations for the treatments. Time to the formation of morula and commencement of hatching (minutes), egg fertilization (%), hatching rate (%), and 96-h larval survival rate (%), were monitored. Microscopy was with Olympus binocular and Carl Zeiss stereo (dissecting) microscopes, aided by camera attachment with a zoom lens (14MP 1080P HDMI c-mount) (Shenzhen Hayear Electronics, Guangdong, China) (total magnification × 400).

Thirty minutes after placement in the incubation units, eggs were washed free of herbicides and incubated in borehole water. Three eggs sampled from each incubation unit were placed in petri dishes and continuously observed for details of the progression of embryogenesis. Periodically, eggs in the incubation units were examined to confirm the observations. Progression of embryogenesis was expressed as time (hours postfertilization) (hpf) to morula formation (end of cleavage) and to commencement of hatching. Time to commencement of hatch was defined as hpf to the attainment of a 3% hatching rate of eggs in the incubation units. The hatching rate was determined by expressing the number of hatchlings at 36 hpf as a percentage of fertilized eggs. Early-stage delineation was according to Ofor and Oke [25].

### 2.4. Oxidative Stress Markers in Tissue Homogenates

Levels of ROS and SOD were determined in 50 freshly striped eggs. Eggs were then activated by placement of the egg–sperm mix in water (control) or fertilizer solution for incubation. Samples of 50 eggs taken at 4 hpf and 8 hpf and larvae taken at 24 hpf and 96 hpf were analyzed to detect ROS and SOD in control and Treatments 1, 3, and 5. Fluorometric methods simultaneously quantify several ROS and are preferred in ROS assay [26]. In this study, a modified oxygen radical absorption capacity (ORAC) method of Huang et al., [27] was used. The method was modified by utilizing the ROS–scavenger butylated hydroxytoluene (BHT) for reader injector priming and as reaction initiator, as well as by the control of the plate reader by Gen5 Data Analysis software (BiotTek Instruments, Inc., Winooski, Vermont) [28].

Spectrophotometric methods are in use for the quantification of antioxidant enzymes [26]. SOD activity was assayed by its ability to inhibit the autooxidation of epinephrine according to Sun and Zigman [29]. Absorbance was read at regular intervals of 1 minute at 480 nm.

### 2.5. Water Quality

Dissolved oxygen (mg L^−1^), pH, and temperature (°C), were measured with a Milwaukee portable DO meter and Hanna digital pH meter model HI98107, respectively.

### 2.6. Data Collection and Analysis

Residuals of data on egg fertilization, time to morula formation and commencement of hatching, hatching rate after 36 hpf, and 96-h larval survival rate were subjected to normality (Shapiro–Wilk's) and homoscedasticity (Levene's) tests. Only fertilization and hatching rates met the criteria and were analyzed to determine differences between treatment means using one-way ANOVA. A pairwise comparison of treatments 0 vs. 1, 0 vs. 2, 0 vs. 3, 0 vs. 4, and 0 vs. 5 was performed using the Tukey HSD post hoc test. The least concentration with a significantly higher effect than a control for each herbicide was determined and designated as the lowest significant effect concentration (LSEC). A Kruskal–Wallis H test of nonparametric data was used to determine differences between mean ranks. Dunn's post hoc test was used for pairwise comparison of treatments 0 vs. 1, 0 vs. 2, 0 vs. 3, 0 vs. 4, and 0 vs. 5. Deviations of embryogenesis and other early-stage endpoints in solutions of each GBH from their control values were calculated. The resulting data did not meet the normality criteria. Mean ranks of treatments were analyzed using independent samples K.–W. H test, followed by Dunn's post hoc with Bonferroni correction for multiple comparisons. From this, the toxicity ranking of the GBHs on the early-stage indices was determined. The larval mortality record was subjected to probit analysis to determine 96-h LC_50_ of the respective GBHs and associated glyphosate concentrations. The toxicity of the GBHs was classified on the basis of their 96-h LC_50_ [30]. The LSEC, ranking, 96-h LC_50_, and toxicity classification were used to rate the aquatic toxicity and environmental risk of application.

Mann–Whitney *U* test was performed to compare levels of ROS and SOD in freshly stripped eggs with levels in eggs in control and treatments 1, 3, and 5 of the various herbicides. The lowest concentration with a significantly higher effect than freshly stripped eggs for each herbicide was determined and designated as LSEC. Statistical analysis of oxidative stress markers was performed on pooled values from samples taken at 4, 8, 24, and 96 hpf. All statistical analyses were performed using SPSS.

## 3. Results

### 3.1. Effect of Herbicide Concentration

**Figure 1:**
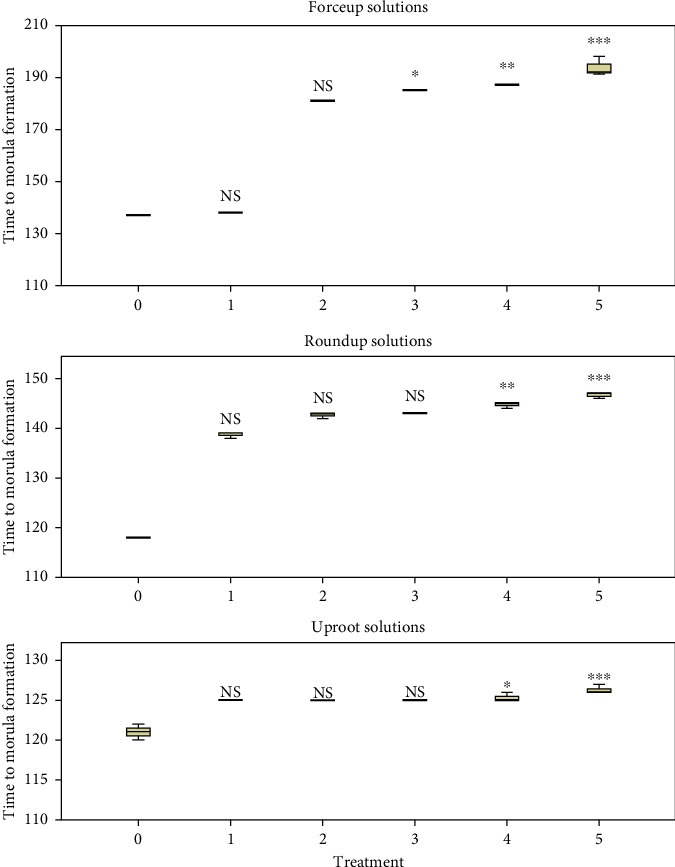
Progression of embryogenesis of fertilized *Clarias gariepinus* eggs to morula formation (minutes) showing a significant effect of 0.006%, 0.013%, 0.025%, 0.05%, and 0.10% (v/v) (Treatments 1, 2, 3, 4, and 5, respectively) concentrations of Forceup, Roundup, and Uproot herbicides ([*χ*^2^] 5 = 15.296, *p* = 0.009) (independent samples Kruskal–Wallis test), in comparison to control (0.0% [v/v]) (Dunn's post hoc test), and the lowest significant effect concentration at 0.025% (v/v) in Forceup and 0.05% (v/v) in Roundup and Uproot herbicides. ^∗^*p* < 0.05, ^∗∗^*p* < −0.01, and ^∗∗∗^*p* < 0.001. NS: not significant (*p* > 0.05). Data points are median ± interquartile range. Treatments in each herbicide were replicated three times. Time to morula formation in each treatment was averaged from three eggs per replicate.

**Figure 2:**
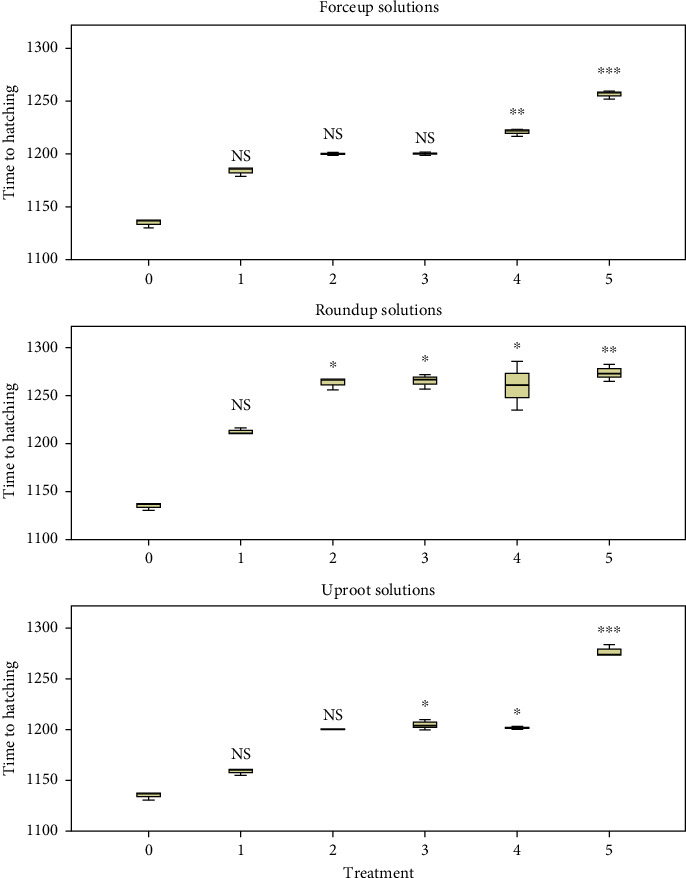
Postcleavage embryogenesis of *Clarias gariepinus* eggs to 3% hatching (minutes) showing a significant effect of 0.006%, 0.013%, 0.025%, 0.05%, and 0.10% (v/v) (Treatments 1, 2, 3, 4, and 5, respectively) concentrations of Forceup, Roundup, and Uproot herbicides ([*χ*^2^] 5 = 12.634, *p*=0.027) (independent samples Kruskal–Wallis test), in comparison to control 0.0% (v/v) (Dunn's post hoc test), and the lowest significant effect concentration at 0.05% (v/v), 0.013% (v/v), and 0.025% (v/v) in Forceup, Roundup, and Uproot herbicides, respectively. ^∗^*p* < 0.05, ^∗∗^*p* < −0.01, and ^∗∗∗^*p* < 0.001. NS: not significant (*p* > 0.05). Data points are median ± interquartile range. Treatments in each herbicide were replicated three times. Time to 3% hatching in each treatment was averaged from replicates.

Comparison of time to the formation of morula between concentrations within herbicides using the K.-W. H test is given in Figure 1. Concentration had a significant effect on Forceup ([*χ*^2^] 5 = 16.928, *p* = 0.005), Roundup ([*χ*^2^] 5 = 16.621, *p* = 0.005), and Uproot ([*χ*^2^] 5 = 15.296, *p* = 0.009) solutions. The comparison of time to commencement of hatching between concentrations within herbicide is shown in Figure 2. Concentration had a significant effect on Forceup ([*χ*^2^] 5 = 16.189, *p* = 0.006), Roundup ([*χ*^2^] 5 = 12.634, *p* = 0.027), and Uproot ([*χ*^2^] 5 = 15.751, *p* = 0.009) solutions.

**Figure 3:**
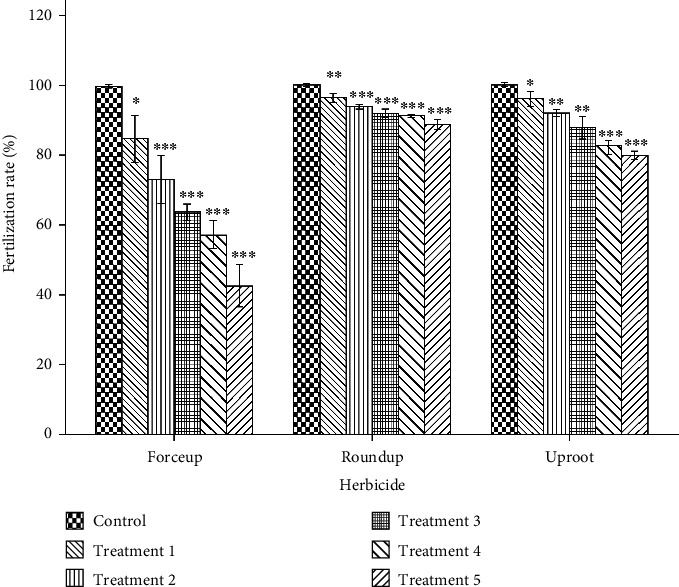
Fertilization rate of *Clarias gariepinu*s eggs (%) in 0.006%, 0.013%, 0.025%, 0.05%, and 0.10% (v/v) concentrations (Treatments 1, 2, 3, 4, and 5, respectively) of Forceup, Roundup, and Uproot solutions used as activation media, showing a significant effect of concentration (*p* < 0.001) (one-way ANOVA). The lowest significant effect concentration in comparison to control (0.0% [v/v]) (Tukey's HSD post hoc multiple comparison) was 0.006% (v/v) in all three herbicides. ^∗^−0.05, ^∗∗^*p* < 0.01, and ^∗∗∗^*p* < 0.001. Bars are means (±SD). Treatments in each herbicide were replicated three times.

**Figure 4:**
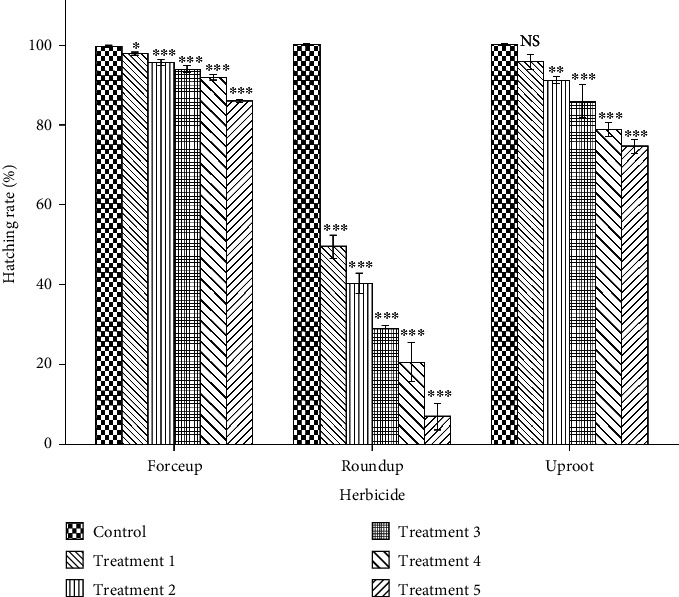
Hatching rate (number of hatchlings expressed as percentage of fertilized eggs) of *Clarias gariepinus* eggs in 0.006%, 0.013%, 0.025%, 0.05%, and 0.10% (v/v) concentrations (Treatments 1, 2, 3, 4, and 5, respectively) of Forceup, Roundup, and Uproot solutions used as activation media showing a significant effect of concentration (*p* < 0.001) (one-way ANOVA). The lowest significant effect concentration in comparison to control (0.0% [v/v]) (Tukey's HSD post hoc multiple comparison) was 0.006% (v/v) in Forceup and Roundup and 0.013% (v/v) in Uproot. ^∗^−0.05, ^∗∗^*p* < 0.01, and ^∗∗∗^*p* < 0.001. Bars are means (±SD). Treatments in each herbicide were replicated three times.

**Figure 5:**
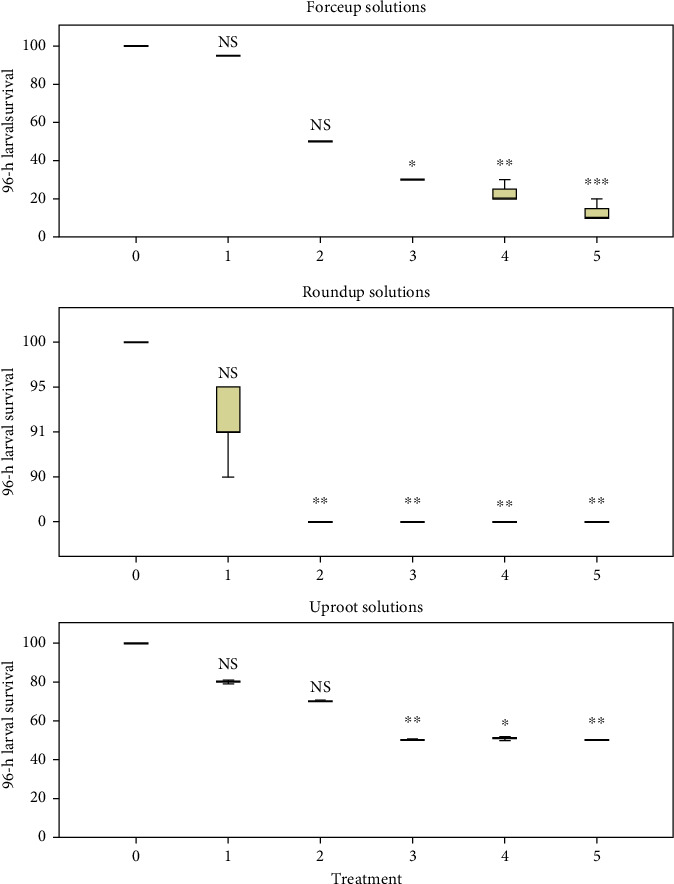
Survival of *Clarias gariepinus* larvae at 96 h in 0.006%, 0.013%, 0.025%, 0.05%, and 0.10% (v/v) solutions of Forceup, Roundup, and Uproot herbicides (Treatments 1, 2, 3, 4, and 5 respectively) showing significant effect of treatment ([*χ*^2^] 5 =15.107, *p*=0.010) (independent samples Kruskal–Wallis test) in comparison to control (0.0% [v/v]) (Dunn's post hoc test). The lowest significant effect concentration was 0.025% (v/v) in Forceup and Uproot and 0.013% (v/v) in Roundup herbicides. ^∗^*p* < 0.05, ^∗∗^*p* < 0.01, and ^∗∗∗^*p* < 0.001. NS, not significant (*p* > 0.05). Data points are median ± interquartile range. Treatments in each herbicide were replicated three times.

Variations in fertilization, hatching, and 96-h survival rates are shown in Figures 3, 4, and 5, respectively. Fertilization and hatching rates were significantly affected by the concentration of each herbicide (*p* < 0.001). Concentration had a significant effect on 96-h survival rates in Forceup[*χ*^2^] 5 = 16.555, *p* = 0.005), Roundup ([*χ*^2^] 5 = 16.900, *p* = 0.005), and Uproot ([*χ*^2^] 5 = 15.107, *p* = 0.010) solutions. Morula formation and hatching were delayed in the herbicide solutions (Table 1). The LSEC of the herbicides on the various toxicity endpoints as well as the spread of data are given in Table 2.

### 3.2. Effect of Herbicide Formulation

The results of the K.-W. H. test of comparison of time to morula formation and to commencement of hatching, fertilization rate, hatching rate, and 96-h larval survival are given in Table 3. Dunn's pairwise test with Bonferroni adjustment for multiple comparisons is also given in Table 3. Forceup was the highest-ranked effect on time to morula formation and fertilization rate. Roundup had the highest-ranked effect on hatching, hatching rate, and 96-h larval LC_50_. Zero percent larval survival was seen in Roundup from Treatment 2 (Figure 5). Uproot had the leastranked effect for all variables (Table 3). The 96-h larval LC_50_ of the herbicides and toxicity classification of the herbicides are given in Table 4.

### 3.3. Oxidative Stress Indices

**Figure 6:**
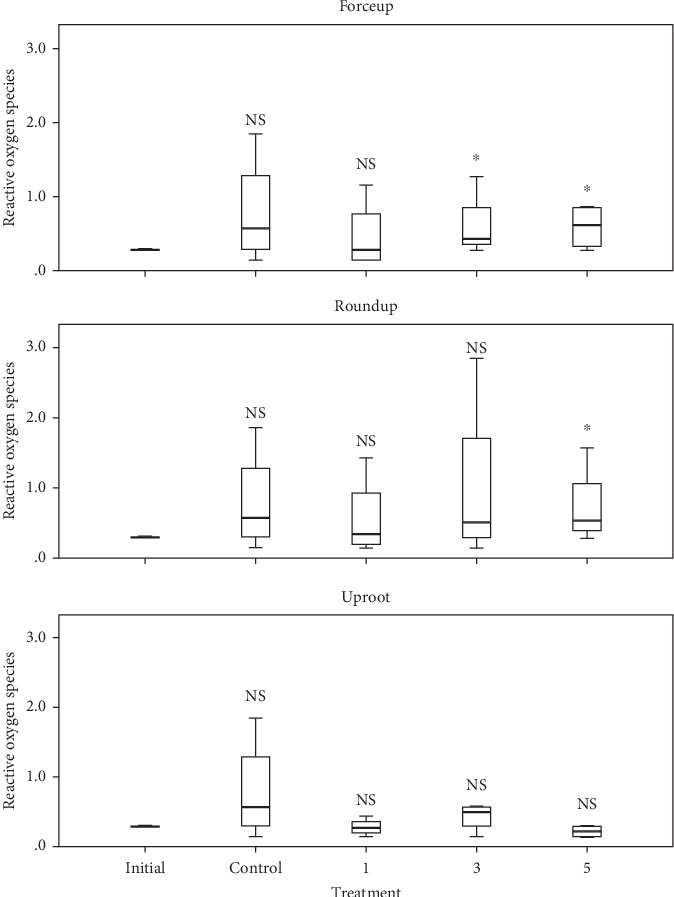
Reactive oxygen species (ROS) levels (U mg^−1^ protein) in early stages of *Clarias gariepinus* in 0.006%, 0.025%, and 0.10% (v/v) (Treatments 1, 3, and 5, respectively) microdilutions of Forceup, Roundup, and Uproot herbicides compared to levels in freshly-stripped eggs. Control: no herbicide, initial = freshly-stripped eggs. ROS levels in embryos in Treatment 5 of Roundup solutions and Treatments 3 and 5 of Forceup solutions were significantly higher than in freshly stripped eggs (^∗^*p* < 0.05) (Mann–Whitney *U* test). Treatments in each herbicide were replicated three times. Values are means from 50 eggs/larvae sampled from each replicate at intervals of 4-, 8-, 24-, and 96-h postfertilization.

**Figure 7:**
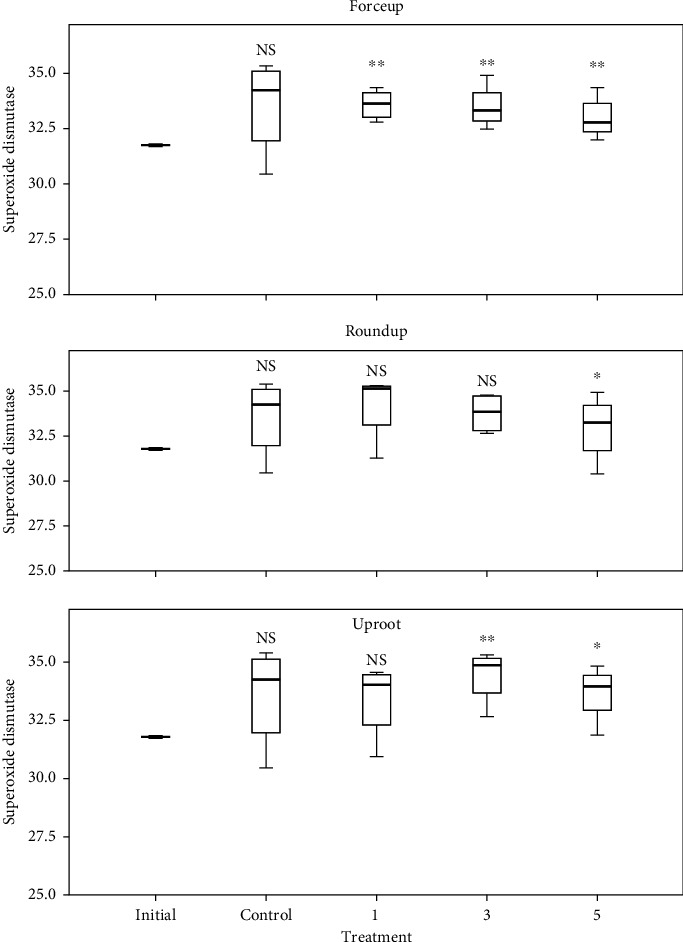
Superoxide dismutase (SOD) levels (U mg^−1^ protein) in early stages of *Clarias gariepinus* in 0.006%, 0.025%, and 0.10% (v/v) (Treatments 1, 3, and 5, respectively) microdilutions of Forceup, Roundup, and Uproot herbicides compared to levels in freshly-stripped eggs. Control: no herbicide, initial = freshly-stripped eggs. SOD levels in embryos in Treatment 5 of Roundup solutions and Treatments 3 and 5 of Forceup and Uproot solutions were significantly higher than in freshly stripped eggs (^∗^*p* < 0.05 and ^∗∗^*p* < 0.01) (Mann–Whitney *U* test). Treatments in each herbicide were replicated three times. Values are means from 50 eggs/larvae sampled from each replicate, at intervals of 4-, 8-, 24-, and 96-h postfertilization.

ROS and SOD levels in embryos in control had a significant correlation (*r* = 0.993, *p* < 0.001). The levels of ROS and SOD in freshly stripped eggs, control, and Treatments 1, 3, and 5 of each herbicide are shown in Figures 6 and 7, respectively. There were no significant differences in ROS levels in Forceup solutions ([*χ*^2^] 4 = 4.82, *p* = 0.307), while differences in levels of SOD were significant ([*χ*^2^] 4 = 10.36, *p* = 0.035). There were no significant differences in ROS levels in Roundup solutions ([*χ*^2^] 4 = 5.090, *p* = 0.278), while differences in levels of SOD were significant ([*χ*^2^] 4 = 10.054, *p* = 0.040). Uproot solutions had significant differences in levels of ROS ([*χ*^2^] 4 = 13.898, *p* = 0.008) and SOD ([*χ*^2^] 4 = 9.765, *p* = 0.045). The lowest concentration of herbicides inducing significant differences in levels of ROS and SOD in embryos in comparison to freshly stripped eggs is shown in Table 2. ROS and SOD in freshly stripped eggs did not significantly vary with levels in control (*p* > 0.05). ROS and SOD had a significant positive correlation (*r* = 0.577, *p* < 0.0.001) across Forceup solutions, Roundup, and Treatments 1 (*r* = −0.667; *p* < 0.007), 3 (*r* = 0.518; *p* < 0.048), and 5 (*r* = 0.847; *p* < 0.001) but no correlations in Uproot solutions.

### 3.4. Water Quality

Control DO levels and pH were 6.69 ± 0.02 mg L^−1^ and 6.9 ± 0.02, respectively. Oxygen levels ranged from 6.40 ± 0.01 mg L^−1^ to 2.95 ± 0.01, 6.69 ± 0.02 to 3.13 ± 0.05, and 6.66 ± 0.02 to 3.09 ± 0.01 mg L^−1^, respectively, in Concentrations 1–5. pH values ranged from 6.40 ± 0.01 to 4.13 ± 0.06, 6.42 ± 0.01 to 5.0 ± 0.0, and 6.41 ± 0.01 to 4.50 ± 0.01 in Roundup, Forceup, and Uproot solutions, respectively. DO and pH were significantly affected by treatment (*p* < 0.001). Temperature ranged from 27.1 ± 0.1 to 27.2 ± 0.0°C and did not significantly vary with treatment (*p* > 0.05).

## 4. Discussion

### 4.1. Toxicity Within Herbicides

There were concentration-dependent toxic effects of the herbicides on *C. gariepinus* egg fertilization rate, embryogenesis, hatching rate, and larval survival rate at microconcentrations of the herbicides tested. The toxic effects increased with an increase in the strength of the microconcentrations. This is important because on the basis of the glyphosate content, toxic effects were seen at microconcentrations lower than the ecological trigger value of glyphosate [24]. On the basis of full formulation, the microconcentrations were remarkably lower than the concentration of the herbicides that would be found in water in which the herbicides have been used according to the recommended procedure. The recommended application rate of Roundup Proactive (360 g/L glyphosate as 441 g/L [35% w/w] of the potassium salt of glyphosate) in enclosed waters, open waters, and land immediately adjacent to aquatic areas, targeting emergent weeds, e.g., reeds, grasses, and watercress, is 5 L/ha ([31] https://agrigem.b-cdn.net/product_downloads/tmtznlr28p.pdf). When this surface area–based rate is volume-converted assuming a water depth of 1.5 m, the resulting volume-based concentration is 0.32% (v/v) of the full formulation. This is higher than the herbicide LSECs observed in this experiment. These results signal an increased potential for the ecotoxic effect of the herbicides. Furthermore, both isopropyl amine salt and potassium salt glyphosate formulations are considered to have low toxicity [32]. Bringer et al. [33] reported similarly low LSEC of 0.11 μg L^−1^ and 0.3 μg L^−1^ for glyphosate and Roundup solutions, respectively, used as incubation media for *Crassostrea gigas* larvae.

Herbicide toxicity in early-stage development was seen in abnormal embryogenesis, expressed as delayed progression in comparison to control (Table 1). Available findings on the implications of delayed cleavage indicate that it is a predictor of future developmental abnormality. This is the case in the Haddock, *Melanogrammus aeglefinus*, for which Rideout, Trippel, and Litvak [34] reported an inverse relationship between hatching success and abnormal cleavage. Zhang, Mutsukawa, and Onozato [35] reported a high correlation (*r* = 0.96) between the frequency of occurrence of delayed cleavage embryos and tetraploidization in heat- and hydrostatic pressure-shocked rainbow trout *Oncorhynchus mykiss* embryos. Heat- and hydrostatic pressure-shocked embryos were at the 4-cell stage, while control embryos were all at the 8-cell stage. Alix et al. [36] reported that delayed cleavage affects reproductive success. Additional indicators of toxic effect were the declining egg fertilization rate, hatching rate, and larval survival rate (Figures 1, 2, 3, 4, and 5). It was also seen in the unhindered increases in ROS of early stages exposed to herbicide microconcentrations in comparison to freshly stripped eggs, indicating oxidative stress in exposed embryos, which had LSEC at Treatment 3 of Forceup solutions and Treatment 5 of Roundup solutions, while no Uproot solution had a significant influence on ROS levels (Figure 6 and Table 2). This was despite significant increases in SOD levels in treatments over freshly stripped eggs, with SOD LSEC at Treatments 1, 5, and 3, respectively, in Forceup, Roundup, and Uproot solutions (Figure 7 and Table 2). On the other hand, ROS and SOD levels in embryos in control solutions increased over levels in freshly stripped eggs. Nevertheless, the increases were not significant, suggesting improved oxidative stress management, leading to normal early-stage development in the control. However, the unimpeded increases in ROS in embryos exposed to the herbicide solutions indicate impairment of oxidative stress management, leading to the observed abnormalities in early-stage development. Embryonic survival is positively related to egg fertilization success [37].

The progression of embryogenesis in control in the present study was normal for the species [38, 39]. Values for fertilization rate, hatching rate, and larval mortality in the control were also normal for the species. These suggest a balance between ROS and SOD defense mechanisms in the control, due to the absence of herbicide toxicity. According to Kadomura et al. [40], the larvae of six marine species, the devil stinger ray (*Inimicus japonicus*), marbled rockfish (*Sebastiscus marmoratus*), black rockfish (*Sebastes inermis*), seven-band grouper (*Epinephelus septemfasciatus*), tiger puffer (*Takifugu rubripes*), and red seabream (*Pagrus major*), produce ROS and SOD under normal rearing conditions and without additional stimuli.

Rapid tissue growth occurs during the early stages of fish development and results in high oxygen consumption [41]. ROS normally results from aerobic metabolism but becomes more significant during this stage [41]. SOD is considered a first line of defense against oxidative stress in aerobic organisms, a function it performs by catalyzing the dismutation of ROS (Shrikanth et al., 2023). Oxidative stress disrupts the balance between the production of ROS and antioxidant defenses and could lead to tissue injury [42]. This disruption, which was clearly evident in embryos exposed to herbicide solutions, but absent in control, diminishes the capacity of the embryo for development [43].

### 4.2. Comparative Effect of Herbicides

Formulation-dependent effect of the herbicides on egg fertilization rate, embryogenesis, hatching rate, and 96-h larval survival rate were also seen at the various microconcentrations tested (Table 3). Among the three herbicides, Forceup had the most toxic effect on egg fertilization and cleavage (Tables 3 and 4). *C. gariepinus* is externally fertilizing. Both eggs and sperm are exposed to the toxic effects of herbicides. Rideout, Trippel, and Litvak [34] reported that fertilization success is a predictor of egg and embryo quality. Lopes et al. [44] reported significant reductions in motility, motility duration, mitochondrial functionality, and membrane and DNA integrity of sperm of *Danio rerio* exposed to glyphosate, resulting in a reduced fertility rate. The use of the solutions as fertilization media exposed the sperm to herbicides. Thus, in addition to the toxic effect on eggs, the effect of herbicides on fertilization may have also been mediated through their effect on sperm. The influence on sperm, egg, and embryo quality is thus environmentally relevant for the reproductive success of *C. gariepinus* as an externally fertilizing species. Forceup had the most toxic effect on fertilization rate in comparison to Roundup and Uproot (Table 3). However, Roundup had the most toxic effect on postcleavage embryogenesis, hatching rate, and survival rate in comparison to Forceup and Uproot (Table 3), with Treatment 2 of Roundup recording 0% survival.

Lugowska [45] reported that Roundup caused a concentration-dependent reduction of common carp embryonic survival but had no effect on the development rate. Webster et al. [46] and Liu et al. [9] reported that Roundup solutions caused premature hatching in zebrafish. The 96-h LC_50_ of Forceup, Roundup, and Uproot reported in this study are 0.02%, 0.005%, and 0.06% concentrations, respectively. These contain 0.08 mg L^−1^, 0.018 mg L^−1^, and 0.22 mg L^−1^ of glyphosate, respectively. The 96-h LC_50_ classify Uproot as “highly toxic,” and Forceup and Roundup as “very highly toxic” [30] (Table 3). In combination with other endpoints in this study, the resulting aquatic toxicity of the herbicides is in the order Uproot < Forceup < Roundup. Since the three herbicides have identical glyphosate levels of 360 mg L^−1^, the differences in toxicity are therefore due to formulation-based differences [3]. The dangers posed by the application of the herbicides in aquatic or riparian environments vary accordingly [5, 10]. The aquatic nontarget impact is a concern in herbicide use [47]. Of recent, low toxicity thresholds of GBHs of 24-h LC_50_ of 0.022 mg L^−1^ and 48-h LC_50_ of 0.0008 mg L^−1^ have been reported [48]. These are similar to the microconcentrations in the present study. The implication of the low concentrations at which these effects manifest is an elevated potential risk of a negative impact on the critical control points in the reproductive process of *C. gariepinus*, including the off-target impact of their application. This is especially because as both the glyphosate trigger value specified by regulation (0.37 mg.L^−1^) [24] and the resulting concentration of the full formulations when used in riparian and aquatic environments (0.32% [v/v]; Roundup Proactive directions for use, 2016: https://agrigem.bcdn.net/product_downloads/tmtznlr28p.pdf) are higher than the LSECs and 96-h LC_50_ reported in this study. The off-target impact of herbicide application may result from spray drift, runoff, and other means of chemical trespass. There is therefore, a need for cautious use of the herbicides in aquatic and riparian environments. Furthermore, as the risk of application varied between herbicides, their usage should be selective use, balancing effectiveness as herbicides, against the risk of environmental toxicity.

## 5. Conclusions

Roundup and Forceup herbicides were more toxic effect on the early-stage indices, as well as oxidative stress, than Uproot. Roundup was by far the most toxic of the three herbicides, with a larval 96-h LC_50_ four times higher and 12 times lower than Forceup and Uproot, respectively. At the microconcentrations tested, the herbicides Roundup, Forceup, and Uproot exerted oxidative stress that contributed to their respective controlling effects on *C. gariepinus'* early-stage development. The toxic effect on the early stages of *C. gariepinus*, an ecologically important species, was also seen at concentrations lower than the glyphosate regulatory trigger value, as well as at the concentrations resulting in aquatic environments when some full formulations are used according to the recommended procedure for such environments. The herbicides may thus have a greater potential for aquatic toxicity than presently recognized. As the risk to reproduction success of *C*. *gariepinus* varies among the GBHs tested, there is a need for selective use of the herbicides, balancing effectiveness as herbicide with the risk of environmental toxicity. The use of GBHs in freshwater aquatic and riparian environments should be minimized or cease completely, in the light of the results of the present study.

## Figures and Tables

**Table 1:** Progression of embryogenesis (time to formation of morula and to hatching) (minutes) of *Clarias gariepinus* eggs incubated in 0.006%, 0.013%, 0.025%, 0.05%, and 0.10% (v/v) (Treatments 1, 2, 3, 4, and 5, respectively) solutions of Forceup, Roundup, and Uproot used as incubation and activation media in comparison to control ([0%] v/v).

Stage of embryogenesis	Herbicide	Outcome^†^ (treatments)			Significance^‡^
		Accelerate	Delay	None	
Morula	Forceup	—	2, 3, 4, 5	1	(*χ*^2^) 5 = 15.219 (*p* = 0.009)
	Roundup	—	All treatments	—	*χ* ^2^ = 13.95 (*p* = 0.016)
	Uproot	—	All treatments	—	*χ* ^2^ = 15.30 (*p* = 0.009)

Hatching	Forceup	2	1, 3, 4, 5	—	*χ* ^2^ = 16.61 (*p* = 0.005)
	Roundup	—	All treatments	—	*χ* ^2^ = 13.55 (*p* = 0.019)
	Uproot	—	All treatments	—	*χ* ^2^ = 15.75 (*p* = 0.008)

*Note:* The predominant effect of the herbicide microdilutions on embryogenesis was to delay progression. Time to morula formation and hatching in each treatment were averaged from three eggs per replicate.

^†^Indicates significant acceleration or delay of embryogenesis from control (Dun's post hoc test).

^‡^Independent samples Kruskal–Wallis test.

**Table 2:** Median (±interquartile range) of time (minutes) to morula formation and to hatching, reactive oxygen species and superoxide dismutase (U mg^−1^ protein), and 96-h larval survival (%); means (±standard deviation) of fertilization and hatching rates (%) of *Clarias gariepinus* eggs exposed to concentrations of Forceup, Roundup, Uproot herbicides; and lowest concentrations of herbicides eliciting significant differences in the indices, in comparison to control.

	Time to morula formation	Time to hatch	96-h larval survival	ROS	SOD	Fertilization rate	Hatching rate
	Median (±IQR)					Mean (±SD)	
Forceup	183 ± 49	1200 ± 38	40 ± 75%	0.427 ± 0.57	33.24 ± 1.72	70.0 ± 19.5	94.2 ± 4.5
Roundup	143 ± 6.25	1259 ± 57.25	0.00 ± 92%	0.429 ± 44	33.56 ± 2.27	93.6 ± 3.9	41 ± 30.6
Uproot	125 ± 0.25	1200 ± 46.75	61 ± 30.25%	0.285 ± 0.288	34.007 ± 2.24	89.6 ± 7.5	87.7 ± 9.4

*Lowest significant effect concentration*							
Forceup	0.025%	0.05%	0.025%	0.025%	0.006%	0.006%	0.006%
Roundup	0.05%	0.0013%	0.013%	0.10%	0.10%	0.006%	0.006%
Uproot	0.05%	0.025%	0.025%	Nil	0.025%	0.006%	0.013%

*Note:* Time to morula formation, time to hatching, larval survival, and fertilization and hatching rates were monitored in eggs exposed to 0.006%, 0.013%, 0.025%, 0.05%, and 0.10% (v/v) herbicide concentrations and control (0% herbicide). ROS and SOD were monitored in eggs exposed to 0.006%, 0.025%, and 0.10% concentrations. Herbicide concentrations were replicated three times. Embryo development was averaged from three eggs per replicate, while ROS and SOD were determined from a sample of 50 eggs and larvae.

**Table 3:** Comparison of embryogenesis (time to morula formation and hatching) (minutes), fertilization rate (%), hatching rate (%), and 96-h larval survival of *Clarias gariepinus*, in 0.006%, 0.013%, 0.025%, 0.05%, and 0.10% (v/v) concentrations of Forceup, Roundup, and Uproot herbicides (Treatments 1, 2, 3, 4, and 5, respectively).

	Treatment	Kruskal–Wallis test		Post hoc^‡§^
		*χ*2	*p*	
*Morula*				
	1	5.7	0.058	
	2	7.32	0.026	U-R (−20.67) U-F (−34.00^∗^) R-F (−13.33)
	3	7.51	0.023	U-R (−21.00) U-F (−38.00^∗^) R-F (−17.00)
	4	7.39	0.025	U-R (−22.3) U-F (−39.7^∗^) R-F (−17.3)
	5	5.97	0.051	

*Hatching*				
	1	2.38	0.304	
	2	7.20	0.027	F-U (−31.67) F-R (−48.67^∗^) U-R (−17.00)
	3	7.20	0.027	F-U (−15.67) F-R (−30.33^∗^) U-R (−14.67)
	4	3.29	0.193	
	5	7.20	0.027	R-F (−51.00) R-U (−75.67^∗^) F-U (24.67)

*Fertilization rate (%)*				
	1	5.42	0.066	
	2	7.20	0.027	R-U (−1.83) R-F (−20.45^∗^) U-F (−18.42)
	3	7.20	0.027	R-U (−4.21) R-F (−27.91^∗^) U-F (−23.71)
	4	7.20	0.027	R-U (−8.72) R-F (−33.82^∗^) U-F (−25.1)
	5	7.20	0.027	F-U (−13.57) F-R (−71.78^∗^) U-R (−58.21)

*Hatching rate (%)*				
	1	6.49	0.039	F-U (−2.44) F-R (−48.64^∗^) U-R (−46.2)
	2	7.20	0.027	F-U (−4.82) F-R (−55.67^∗^) U-R (−50.85)
	3	7.20	0.027	F-U (−8.39) F-R (−65.47^∗^) U-R (−57.08)
	4	7.20	0.027	F-U (−13.57) F-R (−71.78^∗^) U-R (−58.21)
	5	7.20	0.027	F-U (−11.96) F-R (−79.59^∗^) U-R (−67.64)

*96-h larval survival rate (%)*				
	1	7.02	0.030	F-R (−3.00) F-U (−15.00^∗^) R-U (−12.00)
	2	8.00	0.018	U-F (−20.00) U-R (−70.00^∗^) F-R (−50.00)
	3	8.00	0.018	U-F (−20.00) U-R (50.00^∗^) F-R (−30.00)
	4	7.78	0.024	U-F (−26.67) U-R (−50.00^∗^) F-R (−23.33)
	5	7.78	0.020	U-F (−36.67) U-R (−50.00^∗^) F-R (−13.33)

*Note:* Forceup had the highest-ranked influence on morula formation and fertilization rate. Roundup had the highest-ranked influence on time to hatching, hatching rate, and 96-h larval survival. The treatments in each herbicide were replicated three times. Time to morula formation and hatching in each treatment were averaged from three eggs per replicate. Treatment degree of freedom = 2.

Abbreviations: F, Forceup; R, Roundup; U, Uproot.

^∗^ *p* < 0.05.

^‡^Bonferroni-adjusted for multiple comparisons.

^§^Mean difference in parenthesis.

**Table 4:** Toxicity attributes (ranking, 96-h LC_50_, and aquatic toxicity classification) of Forceup, Roundup, and Uproot using *Clarias gariepinus* embryogenesis (time to morula formation and hatching) (minutes), fertilization rate (%), hatching rate (%), and 96-h larval survival (%) in herbicide concentrations of 0.006%, 0.013%, 0.025%, 0.05%, and 0.10% (v/v).

Toxicity classification^†^	Herbicide ranking						96-h LC_50_	Aquatic
	Embryogenesis		Fertilization rate		Hatching rate	96-h larval survival	Glyphosate^‡^	Full^§^
	Morula	Hatching						
Forceup	1	3	1	2	2	0.08	0.02	Very highly toxic
Roundup	2	1	2	1	3	0.018	0.005	Very highly toxic
Uproot	3	2	3	3	1	0.22	0.06	Highly ecotoxic

*Note:* Forceup and Roundup controlled embryogenesis. Uproot had a relatively mild influence on early-stage indices compared to Forceup and Roundup.

^†^According to USDA [30].

^‡^Based on associated glyphosate concentrations (mg L^−1^).

^§^Based on concentrations of full formulations ([v/v] %).

## Data Availability

The data that support the findings of this study are available from the corresponding author upon reasonable request.
